# Selection and Evaluation of Reference Genes for miRNA Expression Analysis in *Bemisia tabaci* Under Insecticide Tolerance

**DOI:** 10.3389/fgene.2022.899756

**Published:** 2022-05-13

**Authors:** Qinghe Zhang, Bingli Gao, Cheng Qu, Chen Luo, Jinda Wang, Ran Wang

**Affiliations:** ^1^ College of Agriculture, Yangtze University, Jingzhou, China; ^2^ Institute of Plant Protection, Beijing Academy of Agriculture and Forestry Sciences, Beijing, China; ^3^ National Engineering Research Center of Sugarcane, Fujian Agricultural and Forestry University, Fuzhou, China

**Keywords:** *Bemisia tabaci*, miRNA, reference gene, normalization, expression profiles, insecticide tolerance

## Abstract

A growing number of studies have focused on the microRNA (miRNA) expression in *Bemisia tabaci*, one devastating agricultural insect pest of the tropical and subtropical areas for which the primary means of control are insecticides. In studying the genetic underpinnings of insecticide resistance, the choice of stable reference genes for normalizing data plays a key role to acquire unbiased expression profile results from quantitative real-time PCR (qPCR) analysis. Expression profiles of 11 selected reference genes were determined systematically in *B. tabaci* exposure to 11 insecticides. Furthermore, we assessed the stability of all the selected candidates in relation to other variables including sex, tissue type, and developmental stage. Candidate reference gene validation was conducted by analyzing the let-7-5p expression under various experimental treatments. Five programs BestKeeper, NormFinder, geNorm, △Ct, and RefFinder were applied to verify the accuracy of the selected candidates. Our results displayed that the best choices of the selected candidates for pymetrozine, sulfoxaflor, flonicamid, cyantraniliprole, afidopyropen, and deltamethrin treatment were miR-1-3p and miR-100-5p, U6 and miR-100-5p were best for chlorpyrifos and imidacloprid treatments, and U6 and miR-1-3p were best for flupyradifurone and β-cypermethrin treatments. The reference genes miR-624, miR-252, and miR-275 worked best in adult tissues, miR-100-5p and miR-1-3p worked best in either sex, and miR-624 and miR-11 were best to use across developmental stages. Not even one reference gene was found to be suitable for all experimental conditions. Our results contributed to the growing body of the literature on qPCR reference gene selection under various experimental conditions and facilitate further investigation on gene expression changes in *B. tabaci*, resulting from pesticide exposure.

## Introduction

MicroRNAs (miRNAs) are a type of non-coding small RNA, about 18–25 nucleotides (nt) in length, which originate from stem-loop RNA precursors and have been reported to be produced by most living organisms including plants, animals, and bacteria (Bartel, 2004; Lee et al., 2004). miRNAs play key roles in post-transcriptional gene regulation and affect various biological processes in arthropods such as development, host–pathogen interactions, immunity, and metamorphosis (Asgari, 2013; Li et al., 2019). Additionally, miRNAs participate in insecticide resistance by regulating the target gene expression, and recently, a growing number of regulatory functions have involved in insecticide resistance demonstrated in various insect pests (Zhang et al., 2021).

Expression profiling of miRNAs is essential for studying their functions in insect pests. Currently, quantitative RT-PCR (qPCR) has been proven to be one popular method for analyzing the miRNA expression due to the accuracy, sensitivity, specificity, and low costs (Shi and Chiang, 2005). However, because a variety of factors such as template RNA integrity, contamination of genomic DNA, complementary DNA (cDNA) template quality, and reverse transcription reaction efficiency impact the accuracy of qPCR, suitable reference genes must be selected and validated in order to standardize and correct the data to reduce variability introduced by these factors (Huggett et al., 2005). 5SrRNA and U6 snRNA are commonly utilized as reference genes for analyzing the expression of miRNA in insects; however, these genes cannot be consistently appropriate with all treatments (Lim et al., 2011; Xiang et al., 2014). In recent years, screening of reference genes for studies of miRNA in arthropods including *Grapholita molesta*, *Helicoverpa armigera*, *Bombus lantschouensis*, *Plutella xylostella*, and *Galeruca daurica* has been performed and found that the reference genes appropriate for assessing the expression of miRNA rely on the species of insect, developmental stage, and environmental conditions, and sometimes, more than one reference gene is needed to normalize the data on gene expression under various abiotic and biotic conditions (Wang X.-W. et al., 2017; Yang et al., 2017; Dong et al., 2019; Zhang et al., 2019; Wang et al., 2021).

*Bemisia tabaci*, the tobacco whitefly, is known as one devastating piercing-sucking pest around the world, having recently been reported to more than 700 species of plants, including important economical and horticultural crops (Wang X. et al., 2017; Horowitz et al., 2020). Apart from inflicting mechanical damage on plants, *B. tabaci* can transmit over 200 plant viruses during feeding (Wei et al., 2017). Since control of whiteflies was largely dependent on long-term application of chemical agents, *B. tabaci* has developed high or extremely high levels of resistance to most commercialized insecticides (Horowitz et al., 2020). Previously, the screening of suitable reference genes for mRNA in *B. tabaci* had been conducted (Li et al., 2013), and the results were contributed to the studies on mechanisms of insecticide resistance, plant virus–host interactions, chemical sensing, and host preference in whitefly (Wei et al., 2017; Xu et al., 2019; Zeng et al., 2019; He et al., 2020; Wang et al., 2020a,b; Yang et al., 2020). In recent years, several microRNA profiles of *B. tabaci* under different treatments have been published (Wang et al., 2016; Li et al., 2018; Hasegawa et al., 2020), but as an essential precondition, suitable reference genes of *B. tabaci* for analyzing the expression of miRNA have not been selected and evaluated.

In our current work, 11 selected reference genes, including 10 miRNAs (miR-9a, miR-1-3p, miR-11, miR-184, miR-275, miR-100-5p, miR-252a, miR-277, miR-279d, and miR-624) from the *B. tabaci* databases of small RNA sequencing, have been used for the study of selection and evaluation in previous reports (Wang X.-W. et al., 2017; Yang et al., 2017; Dong et al., 2019; Zhang et al., 2019; Wang et al., 2021). With one commonly utilized reference gene, U6 snRNA, the aforementioned 11 candidates were selected to normalize gene expression data for *B. tabaci* with insecticide tolerance to 11 popular insecticides used against whiteflies in the field, including pymetrozine, chlorpyrifos, imidacloprid, flupyradifurone, sulfoxaflor, flonicamid, cyantraniliprole, afidopyropen, avermectin, deltamethrin, and β-cypermethrin, and most of them have been recorded as cases of resistance in *B. tabaci* (Wang et al., 2018a; Wang et al., 2020b,c; Zheng et al., 2021; Wang et al., 2022). The influence of tissue type, sex, and developmental stage was also assessed. The results provided several units of appropriate reference genes for more research on the insecticide-associated gene expression of the whitefly.

## Materials and Methods

### Insects and Chemicals

The susceptible strain of *B. tabaci* (MED-S) was sampled in 2009 from damaged poinsettias in the city of Beijing, China (Pan et al., 2011). All populations were reared on the *Gossypium hirsutum* cotton plants, maintained at temperature 27 ± 1°C, relative humidity 60 ± 5%, and under a photoperiod with 14:10 h light and dark cycle. Insecticides utilized were analytically standardized. Afidopyropen and sulfoxaflor were purchased from Dr. Ehrenstorfer, Germany. Flonicamid, pymetrozine, imidacloprid, β-cypermethrin, cyantraniliprole, deltamethrin, avermectin, flupyradifurone, chlorpyrifos, dimethyl sulfoxide, and Triton X-100 were purchased from Sigma-Aldrich, Shanghai, China.

### Determining LC_50_ Values of Insecticides

All whitefly bioassays were conducted using published methods (Wang et al., 2018b), and statistical analysis of insecticide bioassays to determine the LC_50_ values of the tested insecticides was performed using Polo Plus. (2002). Adult whiteflies were exposed to the LC_50_ level concentrations of pymetrozine, chlorpyrifos, imidacloprid, flupyradifurone, sulfoxaflor, flonicamid, cyantraniliprole, afidopyropen, avermectin, deltamethrin, or β-cypermethrin. The tested whitefly adults were exposed to distilled water as the control. After 48 h, all survivors of the tested whiteflies gathered for the next step of work about insecticide tolerance. All adults used in bioassays were less than 7 days old. Males and females were used in a 1:1 ratio.

### Sample Collection for Reference Gene Validation in Relation to the Developmental Stage, Tissue, and Sex

Five developmental stages of the whitefly (eggs, first and second, third, fourth stage nymphs, and adults) were sampled and put into a tube, respectively. Three replicates with a total of 2500 eggs, 2000 first and second instar nymphs, 500 third instar nymphs, 400 fourth instar nymphs, and 200 adults were sampled. Three kinds of adult tissues including the abdomen, thorax, and head were collected by the dissection of whitefly adults, and three replicates with a total of 1500 heads, 900 thoraces, and 600 abdomens were sampled. For samples of females and males, the sex of whitefly adults was identified under one stereomicroscope, and gender-identical ones were gathered together, and 200 males or females were collected as one sample. In total, 200 survival adults of *B. tabaci* after the LC_50_ treatment of each insecticide were randomly collected, and all the aforementioned samples, after collection, were treated with liquid nitrogen for the extraction of RNA.

### Extraction of RNA, Synthesis of cDNA, and Design of Primers

Total RNA samples were extracted from treatments in the use of TRIzol reagent kit (Invitrogen, United States), according to the instruction of the manufacturer, and the quality of all the RNA samples was assessed *via* agarose gel electrophoresis, and also, an ultraviolet spectrophotometer (Nano-Drop-2000, Thermo Scientific) was utilized to check the concentration and purity of the RNA samples, according to the published method (Yang et al., 2017). Then, with the use of RNA as a template, the steps of reverse transcription were carried out following the specification of the Mir-X miRNA First-Strand Synthesis Kit (TaKaRa, China). A total of 10 miRNAs (miR-1-3p, miR-9a, miR-11, miR-184, miR-100-5p, miR-275, miR-252a, miR-279d, miR-277, and miR-624) and U6 snRNA, one commonly used reference gene, were set as the candidates for evaluation, according to the published database of *B. tabaci* miRNA-seq and our database (Wang et al., 2016; Li et al., 2018; Hasegawa et al., 2020). Primers of the candidate miRNA were designed in utilizing the software of Primer 5.0 based on the specification of the Mir-X miRNA First-Strand Synthesis Kit (TaKaRa, China), and the reverse primer of all miRNAs was a universal reverse primer (mRQ 3′Primer) from the kit.

### Quantitative PCR and Validation of Reference Gene Selection

According to the published method, for measuring the expression of miRNAs (Li et al., 2018), qPCR was conducted using the TB Green^®^ Premix Ex Taq™ II (Tli RNase H Plus) kit (TaKaRa, China). The total mixture of reaction was 20 µL containing 2 µL of cDNA, 10 µL of TB Green Premix Ex Taq II (Tli RNase H Plus), 6.4 µL of distilled water, 0.8 µL reverse primer, and 0.8 µL forward primer. The conditions of qPCR were 95°C for 30 s, followed by 40 cycles of 95°C for 5 s, and 60°C for 34 s, and the method of 2^-△△*Ct*^ was applied to assess the expression of miRNA. A series of 10^n^-fold diluted cDNA were utilized to establish the standard curves of five points to assess the amplification efficiency of qPCR (*E*) and the determination coefficient (*R* ^*2*^) of each primer pair. *E* values were measured on the basis of the equation: *E* = (10^[−1/slope]^-1) × 100 (Radonić et al., 2004). As one target gene, let-7-5p was selected to conduct validation of the candidate reference genes with various treatments. In comparison with the expression of let-7-5p, the most stable gene (NF1), the best combination of genes (NF1–2)/(NF1–3), and the most unstable gene (NF11) were set as reference genes, and then, the expression levels of let-7-5p were measured by the published approach (Livak and Schmittgen, 2001).

### Data Analysis

The stability of expression in 11 selected reference genes was assessed using Internet-based calculating software RefFinder, which includes four programs geNorm, △Ct, NormFinder, and BestKeeper. (Vandesompele et al., 2002; Andersen et al., 2004; Pfaffl et al., 2004; Silver et al., 2006; Xie et al., 2012). In accordance with ranking each program, the software of RefFinder was used to conduct calculation of the qPCR results for the comprehensive order of rank (Xie et al., 2012). Considering that the order of expression stability could not indicate the suitable number to choose for normalizing data, the program of geNorm was utilized to confirm the suitable number that need to be utilized within specific treatments, depending on the values of pairwise variation (V). When V_n/n + 1_ < 0.15, the suitable number is N. Analysis of statistical differences was conducted by the use of student’s *t* test between the most unstable gene and the best combination (SPSS, 2011).

## Results

### Expression Profiles of Candidate Reference Genes

**FIGURE 1:**
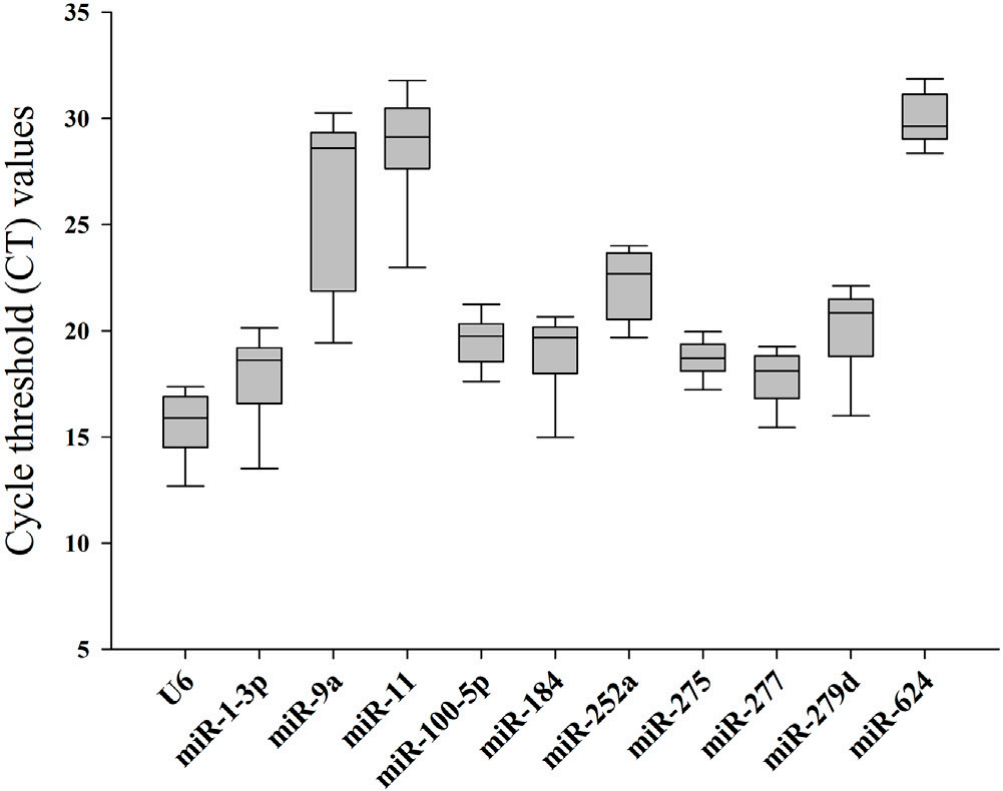
Cycle threshold (Ct) values of candidate reference genes in *Bemisia tabaci*.

In order to obtain standard curves of selected candidates, qPCR tests were carried out. As shown in Table 1, the determination coefficient (*R* ^*2*^) of the 11 selected reference genes was measured (from 0.9564 to 0.9988), demonstrating that the linear relationship between specific working concentrations of the template and their values of Ct was solid. Values of amplification efficiency (*E*) were also calculated (from 94.51% to 113.59%), demonstrating the primers’ effectiveness. As shown in Figure 1 and Table 2, the mean Ct values of the candidates ranged from 15.64 to 29.95, and the Ct SD values ranged from 1.04 to 4.35, indicating that miR-277 and miR-11 displayed the smallest and largest variation, respectively, among all the selected candidates.

**TABLE 1:** Sequences and amplification characteristics of candidate reference genes.

Candidate	Primer sequence (5′→ 3′)	*E* (%)^a^	*R* ^*2*^ ^b^	Slope
miR-1-3p	F: CGC​GTG​GAA​TGT​AAA​GAA​GTA​TGG​AG	98.27	0.9883	−3.364
miR-9a	F: CGC​TCT​TTG​GTA​TTC​TAG​CTG​TAG​GAT	94.51	0.9732	−3.461
miR-11	F: CGC​ATC​ACA​GTC​AGA​GTT​CTA​GCT	108.20	0.9944	−3.144
miR-100-5p	F: AAC​CCG​TAG​ATC​CGA​ACT​TGT​GAA	107.95	0.9930	−3.145
miR-184	F: CGT​GGA​CGG​AGA​ACT​GAT​AAG​GG	97.67	0.9988	−3.379
miR-252a	F: CTA​AGT​ACT​CCG​TGC​CGC​AGG​A	94.54	0.9630	−3.464
miR-275	F: TCA​GGT​ACC​TGA​AGT​AGC​GCG	95.68	0.9598	−3.433
miR-277	F: CGC​TAA​ATG​CAC​TAT​CTG​GTA​CGA​C	103.72	0.9905	−3.236
miR-279 d	F: CGC​GTG​ACT​AGA​TTT​TCA​CTC​ATT​C	107.81	0.9815	−3.148
miR-624	F: CGC​TAT​TCA​CCA​GTA​CTT​GTA​GTC​TCA	103.09	0.9564	−3.251
let-7-5p	F: CCG​CTG​AGG​TAG​TAG​GTT​GTA​TAG​T	113.59	0.9666	−3.034
U6 snRNA	F: Supplied with kit (TaKaRa catalog no. 638313)	97.51	0.9976	−3.384
	R: Supplied with kit (TaKaRa catalog no. 638313)			
mRQ 3′ primer	Supplied with kit (TaKaRa catalog no. 638313)			

a *E*, amplification efficiency.

b *R* ^*2*^, determination coefficient.

**TABLE 2:** Cycle threshold (Ct) values of candidate reference genes in *B. tabaci* under different treatments.

Gene	Ct max	Ct min	Ct difference	Ct mean	Ct SD
miR-1-3p	22.79	11.28	11.51	15.64	2.15
miR-9a	27.95	9.76	18.19	18.26	3.47
miR-11	31.56	16.15	15.41	26.28	4.35
miR-100-5p	33.55	22.15	11.4	28.66	2.84
miR-184	23.69	16.65	7.04	19.60	1.45
miR-252a	22.01	11.28	10.73	18.64	2.39
miR-275	25.15	18.8	6.35	22.24	1.66
miR-277	21.9	16.8	5.1	18.73	1.04
miR-279d	20.57	11.26	9.31	17.60	1.82
miR-624	23.76	9.59	14.17	19.74	2.91
U6	33.28	26.75	6.53	29.95	1.35

### Expression Stability of the Selected Reference Genes Under Insecticide Tolerance and Other Experimental Conditions

**FIGURE 2:**
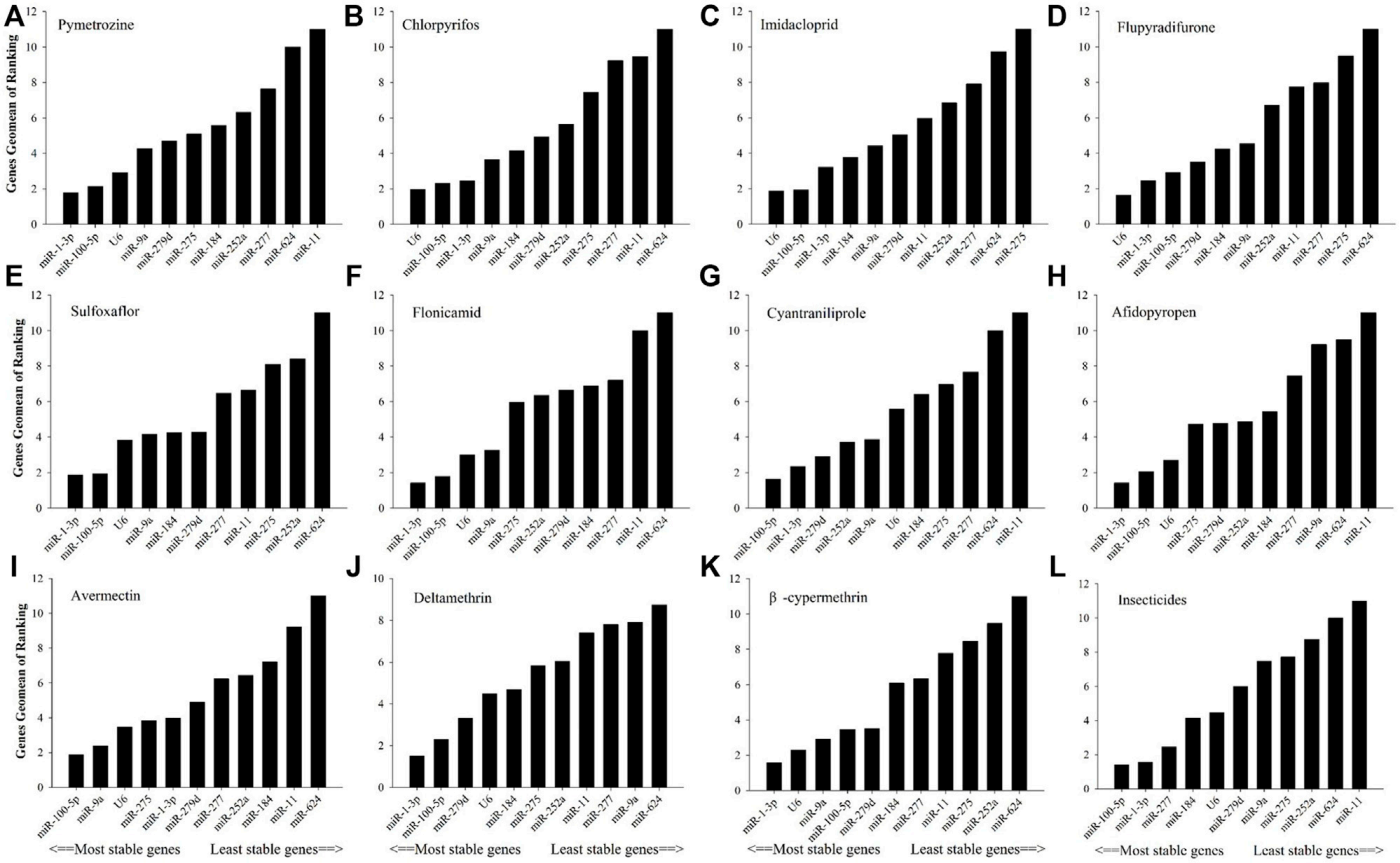
Stability of candidate reference genes in *B. tabaci* under various tolerance for insecticides. In a RefFinder analysis, increasing Geomean values correspond to decreasing gene expression stability. The Geomean values for the following *B. tabaci* samples are presented. **(A)** Pymetrozine: samples treated with pymetrozine; **(B)** chlorpyrifos: samples treated with chlorpyrifos; **(C)** imidacloprid: samples treated with imidacloprid; **(D)** flupyradifurone: samples treated with flupyradifurone; **(E)** sulfoxaflor: samples treated with sulfoxaflor; **(F)** flonicamid: samples treated with flonicamid; **(G)** cyantraniliprole: samples treated with cyantraniliprole; **(H)** afidopyropen: samples treated with afidopyropen; **(I)** avermectin: samples treated with avermectin; **(J)** deltamethrin: samples treated with deltamethrin; **(K)** β-cypermethrin: samples treated with β-cypermethrin; and **(L)** insecticides: samples treated with all tested insecticides. The candidate reference genes are as follows: miR-1-3p, miR-9a, miR-11, miR-184, miR-100-5p, miR-275, miR-252a, miR-279d, miR-277, miR-624, and U6 snRNA.

**FIGURE 3:**
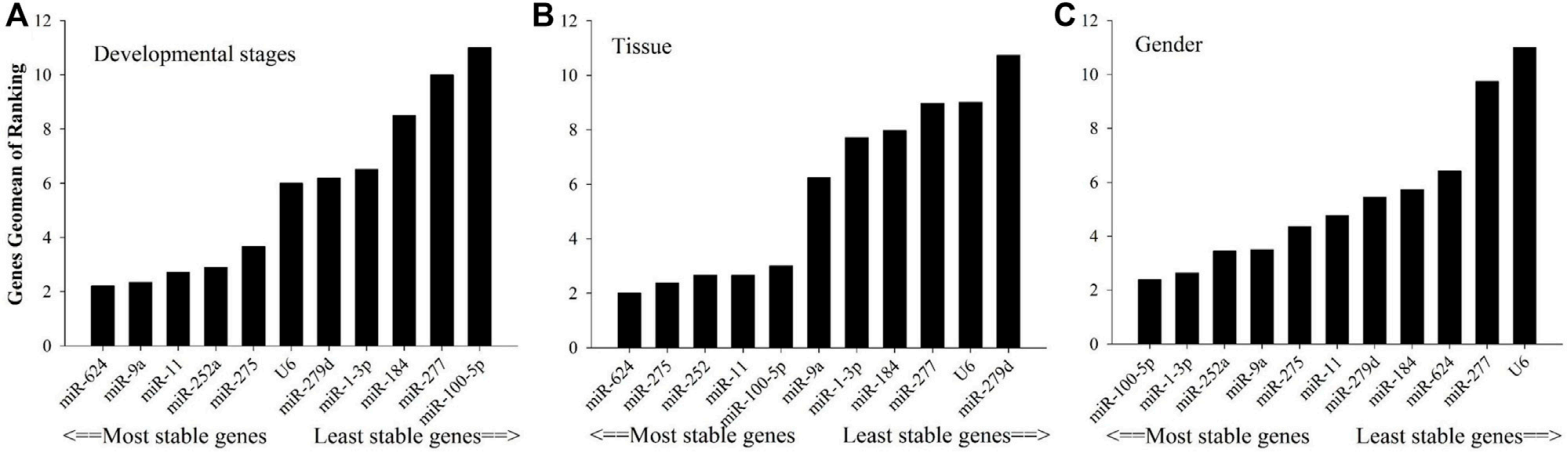
Stability of candidate reference genes in *B. tabaci* under various conditions. In a RefFinder analysis, increasing Geomean values correspond to decreasing gene expression stability. The Geomean values for the following *B. tabaci* samples are presented. **(A)** Developmental stage: samples for all developmental stages; **(B)** tissue: samples for different tissues of adults; and **(C)** adult samples for different genders.

As shown in Figure 2, in relation to effects of insecticide tolerance, △Ct, NormFinder, and geNorm analyses indicated that miR-1-3p, miR-100-5p, and miR-277 were the most stable reference genes, yet BestKeeper evaluated miR-100-5p, miR-184, and miR-1-3p rather than miR-100-5p, miR-1-3p, and miR-277. The results of RefFinder showed the order of rank for stability of genes was from the least to most stable, miR-11, miR-624, miR-252a, miR-275, miR-9a, miR-279d, U6, miR-184, miR-277, miR-1-3p, and miR-100-5p. As shown in Figure 3, for other treatment settings, the most stable reference genes were miR-624 for developmental stages and adult tissues and miR-100-5p for gender, respectively. As shown in Supplementary Table S1, all data on the order of rank for stability of genes are presented in the part of Supplementary Material.

### Optimal Number of Reference Genes

**FIGURE 4:**
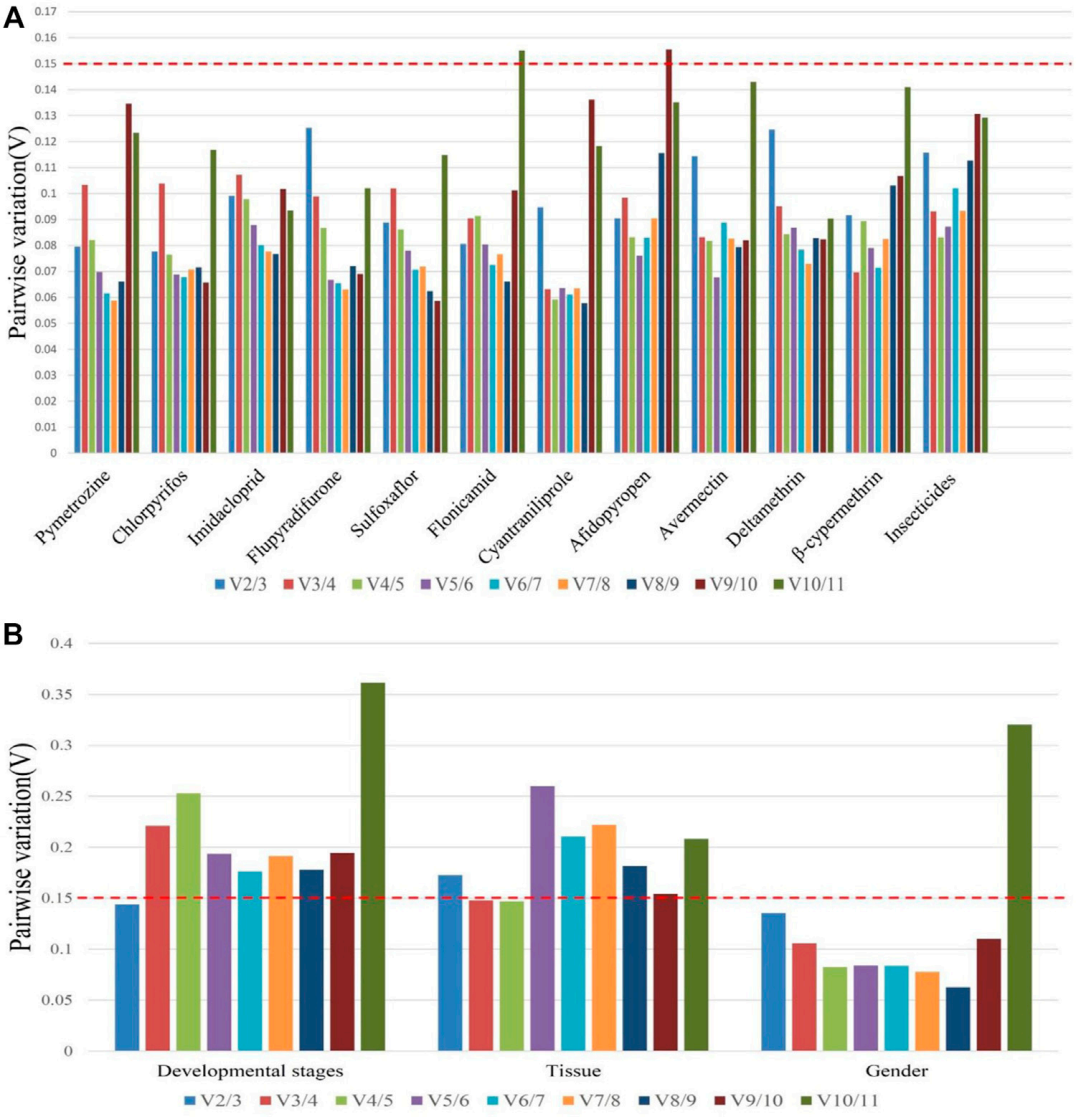
Pairwise variation analysis for an accurate normalization in *B. tabaci*. **(A)** Insecticide tolerance of 11 chemical agents and **(B)** other experimental conditions such as developmental stages, adult tissues, and gender.

Based on the values of the paired variation (Figure 4), the values of V2/3 for all insecticide tolerance, developmental stage, and sex were <0.15 except for in adult tissues, in which the V3/4 value was <0.15. According to the results of overall ranking, the most suitable combinations of reference genes were miR-1-3p and miR-100-5p for insecticide tolerance, miR-100-5p and miR-1-5p for sex, miR-9a and miR-624 for the developmental stage, and miR-624, miR-252, and miR-275 for the tissue type.

### Validation of the Selected Reference Genes

**FIGURE 5:**
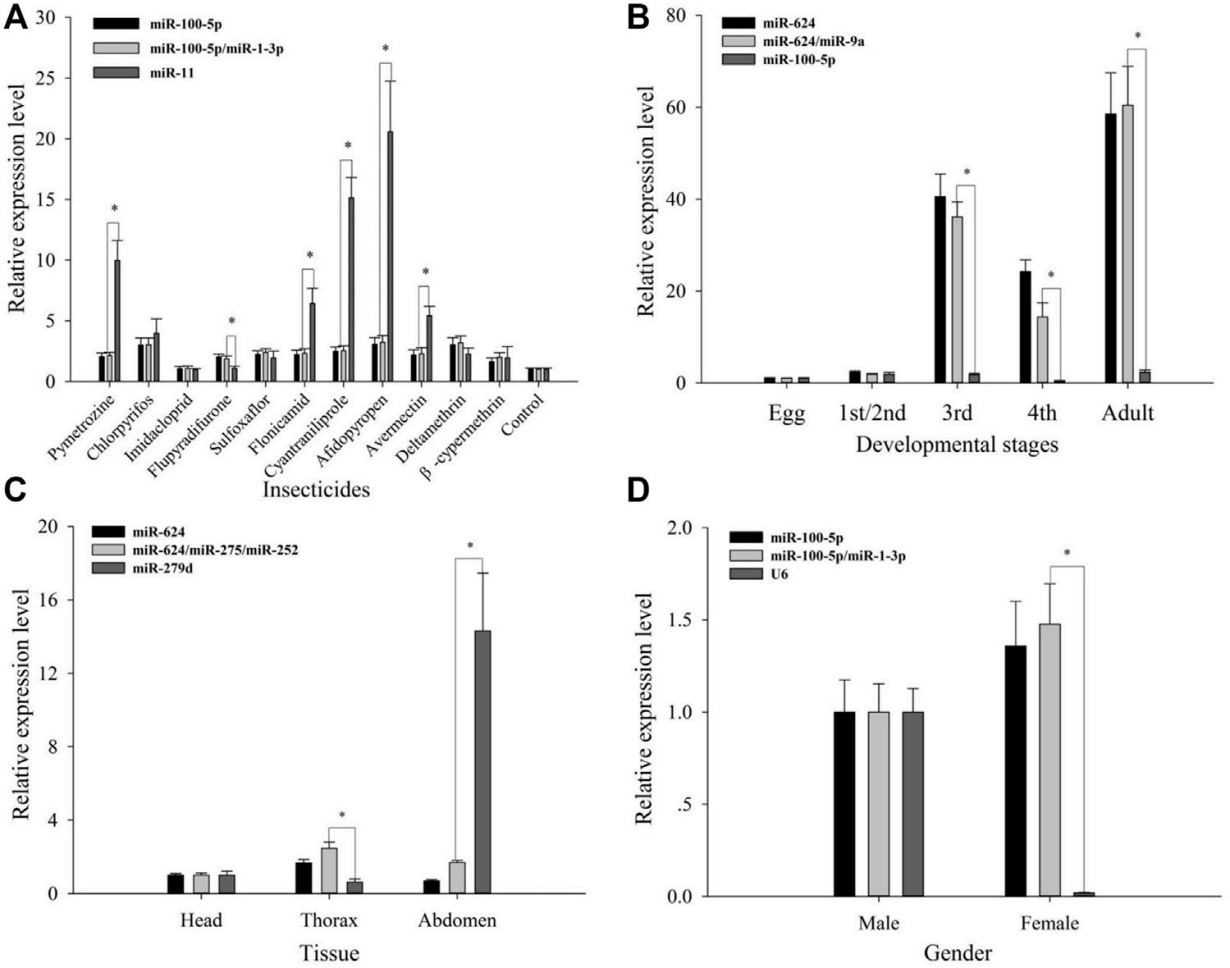
Validation of reference gene selection. Relative expression levels of the target gene let-7-5p under different treatments. **(A)** Insecticide tolerance, **(B)** developmental stages, **(C)** adult tissues, and **(D)** genders. The results are displayed as the mean ± based on three independent biological replicates. The *t*-test was used for statistical difference analysis between the best combination of reference genes and the worst, and statistical differences were denoted by * (p < 0.05).

In order to better verify the reliability of the 11 candidates, let-7-5p was set as one target gene for validating them (Figure 5). Expression profiles of let-7-5p under tolerance for various insecticides were stable, while normalized using NF1 (miR-100-5p) and NF (1–2) (miR-100-5p and miR-1-3p). Moreover, across developmental stages, while by the use of NF1 (miR-624) and NF(1–2) (miR-624 and miR-9a), expression profiles of let-7-5p increased from third instar nymphs to adults, relative to the first two stages, of which a significant difference with the use of NF1 and NF (1–2) was not observed. Similarly, the expression level of let-7-5p in females was higher than that in males while normalized by the use of NF1 (miR-100-5p) and NF (1–2) (miR-100-5p and miR-1-5p), and no significant difference was observed when using NF1 and NF (1–2). Let-7-5p was most highly expressed in the thorax tissue while normalized with the use of NF1 (miR-624) and NF (1-3) (miR-624, miR-252, and miR-275), and the highest expression occurred in the abdomen using NF11 (miR-279d) in comparison with the normalization using NF1 (miR-624) and NF (1-3) (miR-624, miR-252, and miR-275).

## Discussion

A series of published reports have indicated that miRNAs exert regulatory functions in a variety of insects by regulating the expression of target genes (Li et al., 2019; Zhang et al., 2021). Considering that miRNAs account for a very low percentage of the total RNA profile, it is necessary to use accurate and sophisticated methods for analyzing the expression of less abundant miRNAs and exploring their functions (Sokol, 2008). Thus, qPCR has been universally applied to calculate the miRNA expression, and assessment of appropriate reference genes for normalizing results of qPCR is vital to acquire accurate and consistent results. In the current work, 10 miRNAs and one commonly utilized reference gene were selected as candidates and assessed for their ability to reliably normalize the miRNA expression. We utilized the online program RefFinder which includes the four programs Normfinder, BestKeeper, △Ct, and geNorm to identify and rank the best *B. tabaci* reference genes to use with various treatments. Based on the data, we found that different programs gave disparate rankings of candidate reference gene stability under identical treatments and distinct rankings of gene stability under different treatments in an identical program. In *Galeruca daurica*, the best combinations of reference genes were miR-9a-5p and U6 snRNA for different stages of development, miR-305-5p and miR-100-5p for different tissue types, and miR-100-5p and miR-275-3p for various temperature settings (Wang et al., 2021). The best reference gene combinations in *Helicoverpa armigera* were miR-9 and U6 snRNA for stages of development, miR-305 and miR-100 for tissue types, and miR-100 and miR-92a for temperature treatments, and in *Grapholita molesta*, the most suitable combinations were miR-16 and U6 snRNA for the stages of development, and miR-281 and U6 snRNA for different settings of temperature (Wang X. et al., 2017).

Our current work assessed the expression stability of 11 candidates for evaluating reference genes in *B. tabaci* with exposure to 11 pesticides, which are widely used for the management of this destructive insect pest. Furthermore, the stability of the 11 candidates was assessed in various types of adult tissue, stages of development, and sex of *B. tabaci*. Our results indicated that the miR-1-3p expression was highly stable across the insecticides pymetrozine, sulfoxaflor, flonicamid, afidopyropen, deltamethrin, and β-cypermethrin; U6 snRNA was highly stable across the insecticides imidacloprid, flupyradifurone, and chlorpyrifos, and miR-100-5p was highly stable across the insecticides cyantraniliprole and avermectin. Similarly in *H. armigera*, appropriate reference genes were miR-9 and U6 snRNA for insecticide treatment (Yang et al., 2017). For the other tested variables, miR-624 was the most suitable for the stages of development and tissue types and miR-100-5p for sex. In *G. daurica*, miR-100-5p was the most suitable for the tissue type (Wang et al., 2021). Also, U6 snRNA can be utilized as one suitable reference gene for the quantification of miRNA for different stages of development (Feng et al., 2014). Thus, our data further demonstrated that not even one reference gene appropriate for whole treatment of experiments can be found out.

MicroRNA let-7 was initially identified in the nematode *Caenorhabditis elegans*, and it has been found to function in a variety of biological processes in nematodes, flies, and mammals (Lee et al., 2016). To validate the 11 reference gene candidates, the expression levels of let-7-5p in *B. tabaci* were measured and compared with, the most suitable combination and the least suitable reference gene, respectively, with various treatments. Our data indicated that let-7-5p shows higher levels of expression in adults than any other developmental stage and lower levels in eggs. Similarly, in *G. molesta* and *G. daurica*, a higher expression in adults and lower expression in eggs were observed (Wang X.-W. et al., 2017; Wang et al., 2021). Furthermore, it has been demonstrated that inappropriate reference genes can greatly affect miRNA quantification and give rise to inaccurate results, and several publications report similar phenomena in various insect pests, emphasizing the need to assess stable reference genes for reliable determination of the miRNA expression (Wang X. et al., 2017; Yang et al., 2017; Zhang et al., 2019; Wang et al., 2021). In the current work, the expression levels of let-7-5p normalized with the best reference gene, and the best combination of reference genes was similar in each of the tested treatment. Hence, it can be concluded that although the best reference gene is commonly used for normalization in qPCR under most circumstances, it should still be evaluated under new experimental conditions.

## Conclusion

In summary, we assessed the choice of stable reference genes for normalizing data, which plays a key role to acquire unbiased expression profile results from qPCR analysis. Expression profiles of 11 selected reference genes were determined systematically in *B. tabaci* tolerance to 11 insecticides. Furthermore, we assessed the stability of all the selected candidates in relation to other variables including sex, tissue type, and developmental stage. The results contribute to the growing body of the literature on qPCR reference gene selection of miRNA under various experimental conditions and facilitate further investigation on miRNA regulation of gene expression changes in *B. tabaci* associated with insecticide tolerance.

## Data Availability

The original contributions presented in the study are included in the article/Supplementary Materials, further inquiries can be directed to the corresponding authors.
