# Supplementary material for: Selection and Evaluation of Reference Genes for miRNA Expression Analysis in Bemisia tabaci Under Insecticide Tolerance
Source: Front Genet. 2022 May 13;13:899756. doi: 10.3389/fgene.2022.899756 (PMC9136102; doi:10.3389/fgene.2022.899756)
Supplement: Supplementary file 1 [file Table1.DOCX]

Table S1. Expression stability of candidate reference genes under different experimental conditions

| Reference gene | GeNorm M-value | NormFinder Stability | BestKeeper SD value | ΔCt Mean | RefFinder | Ranking Order |
| --- | --- | --- | --- | --- | --- | --- |
| Pymetrozine |  |  |  |  |  |  |
| miR-1-3p | 0.204(1) | 0.189(2) | 0.465(5) | 0.56(1) | 1.78 | 1 |
| miR-100-5p | 0.204(1) | 0.085(1) | 0.637(7) | 0.58(3) | 2.14 | 2 |
| U6 | 0.240(3) | 0.206(3) | 0.464(4) | 0.57(2) | 2.91 | 3 |
| miR-9a | 0.335(4) | 0.527(7) | 0.294(2) | 0.69(6) | 4.28 | 4 |
| miR-279d | 0.415(6) | 0.614(9) | 0.2(1) | 0.76(9) | 4.7 | 6 |
| miR-275 | 0.440(7) | 0.339(4) | 0.56(6) | 0.66(4) | 5.09 | 5 |
| miR-184 | 0.383(5) | 0.591(8) | 0.36(3) | 0.75(8) | 5.57 | 7 |
| miR-252a | 0.467(8) | 0.353(5) | 0.651(8) | 0.68(5) | 6.32 | 8 |
| miR-277 | 0.500(9) | 0.433(6) | 0.751(9) | 0.72(7) | 7.64 | 9 |
| miR-624 | 0.652(10) | 1.085(10) | 1.479(10) | 1.18(10) | 10 | 10 |
| miR-11 | 0.769(11) | 1.227(11) | 1.61(11) | 1.30(11) | 11 | 11 |
| Chlorpyrifos |  |  |  |  |  |  |
| U6 | 0.250(3) | 0.067(1) | 0.694(5) | 0.50(1) | 1.97 | 1 |
| miR-100-5p | 0.231(1) | 0.101(2) | 0.79(7) | 0.50(1) | 2.3 | 2 |
| miR-1-3p | 0.231(1) | 0.202(3) | 0.573(4) | 0.51(3) | 2.45 | 3 |
| miR-9a | 0.380(5) | 0.494(6) | 0.334(1) | 0.65(6) | 3.66 | 4 |
| miR-184 | 0.343(4) | 0.442(5) | 0.514(3) | 0.63(5) | 4.16 | 4 |
| miR-279d | 0.411(6) | 0.512(7) | 0.337(2) | 0.67(7) | 4.92 | 6 |
| miR-252a | 0.445(7) | 0.351(4) | 0.878(9) | 0.61(4) | 5.63 | 7 |
| miR-275 | 0.493(8) | 0.517(8) | 0.723(6) | 0.71(8) | 7.44 | 8 |
| miR-277 | 0.530(9) | 0.531(9) | 0.995(10) | 0.71(8) | 9.24 | 9 |
| miR-11 | 0.574(10) | 0.606(10) | 0.864(8) | 0.71(10) | 9.46 | 10 |
| miR-624 | 0.668(11) | 1.021(11) | 1.481(11) | 1.09(11) | 11 | 11 |
| Imidacloprid |  |  |  |  |  |  |
| U6 | 0.267(1) | 0.133(2) | 0.901(6) | 0.56(1) | 1.86 | 1 |
| miR-100-5p | 0.267(1) | 0.105(1) | 1.02(7) | 0.58(2) | 1.93 | 2 |
| miR-1-3p | 0.306(3) | 0.272(3) | 0.807(4) | 0.61(3) | 3.22 | 3 |
| miR-184 | 0.444(5) | 0.464(5) | 0.653(2) | 0.70(4) | 3.76 | 4 |
| miR-9a | 0.491(6) | 0.590(8) | 0.415(1) | 0.77(8) | 4.43 | 6 |
| miR-279d | 0.385(4) | 0.418(4) | 1.097(8) | 0.70(4) | 5.03 | 5 |
| miR-11 | 0.536(7) | 0.508(6) | 0.879(5) | 0.76(6) | 5.96 | 7 |
| miR-252a | 0.610(9) | 0.689(9) | 0.746(3) | 0.85(9) | 6.84 | 8 |
| miR-277 | 0.572(8) | 0.532(7) | 1.227(10) | 0.76(7) | 7.91 | 9 |
| miR-624 | 0.690(10) | 0.891(10) | 1.161(9) | 1.03(10) | 9.74 | 10 |
| miR-275 | 0.765(11) | 1.002(11) | 1.757(11) | 1.10(11) | 11 | 11 |
| Flupyradifurone |  |  |  |  |  |  |
| U6 | 0.234(1) | 0.181(1) | 0.548(7) | 0.51(1) | 1.63 | 1 |
| miR-1-3p | 0.340(3) | 0.234(2) | 0.262(3) | 0.52(1) | 2.45 | 2 |
| miR-100-5p | 0.234(1) | 0.242(3) | 0.594(8) | 0.53(3) | 2.91 | 3 |
| miR-279d | 0.442(6) | 0.401(5) | 0.125(1) | 0.60(5) | 3.5 | 4 |
| miR-184 | 0.391(4) | 0.242(4) | 0.406(5) | 0.60(4) | 4.23 | 5 |
| miR-9a | 0.428(5) | 0.440(7) | 0.152(2) | 0.61(6) | 4.53 | 6 |
| miR-252a | 0.487(8) | 0.401(6) | 0.506(6) | 0.63(7) | 6.7 | 7 |
| miR-11 | 0.536(9) | 0.701(10) | 0.339(4) | 0.81(10) | 7.75 | 9 |
| miR-277 | 0.462(7) | 0.440(8) | 0.596(9) | 0.633(8) | 7.97 | 8 |
| miR-275 | 0.583(10) | 0.617(9) | 0.827(10) | 0.76(9) | 9.49 | 10 |
| miR-624 | 0.650(11) | 0.870(11) | 0.984(11) | 0.95(11) | 11 | 11 |
| Sulfoxaflor |  |  |  |  |  |  |
| miR-1-3p | 0.163(1) | 0.173(2) | 0.648(6) | 0.50(1) | 1.86 | 1 |
| miR-100-5p | 0.163(1) | 0.161(1) | 0.782(7) | 0.50(1) | 1.93 | 2 |
| U6 | 0.237(3) | 0.240(3) | 0.865(8) | 0.54(3) | 3.83 | 3 |
| miR-9a | 0.427(6) | 0.427(5) | 0.349(2) | 0.61(5) | 4.16 | 4 |
| miR-184 | 0.380(5) | 0.378(4) | 0.581(4) | 0.60(4) | 4.23 | 4 |
| miR-279d | 0.467(7) | 0.495(8) | 0.287(1) | 0.66(6) | 4.28 | 6 |
| miR-277 | 0.328(4) | 0.487(7) | 0.885(9) | 0.67(7) | 6.48 | 7 |
| miR-11 | 0.504(8) | 0.514(9) | 0.497(3) | 0.68(9) | 6.64 | 8 |
| miR-275 | 0.535(9) | 0.456(6) | 0.935(10) | 0.67(8) | 8.11 | 9 |
| miR-252a | 0.564(10) | 0.574(10) | 0.583(5) | 0.73(10) | 8.41 | 10 |
| miR-624 | 0.658(11) | 1.013(11) | 1.398(11) | 1.28(11) | 11 | 11 |
| Flonicamid |  |  |  |  |  |  |
| miR-1-3p | 0.177(1) | 0.221(2) | 0.382(2) | 0.275(2) | 1.41 | 1 |
| miR-100-5p | 0.177(1) | 0.102(1) | 0.56(7) | 0.367(5) | 1.78 | 2 |
| U6 | 0.230(3) | 0.234(3) | 0.527(6) | 0.31(3) | 3 | 3 |
| miR-9a | 0.306(4) | 0.470(7) | 0.408(3) | 0.188(1) | 3.25 | 4 |
| miR-275 | 0.471(7) | 0.402(5) | 0.181(1) | 0.374(6) | 5.96 | 5 |
| miR-252a | 0.546(9) | 0.303(4) | 0.515(4) | 0.604(9) | 6.34 | 6 |
| miR-279d | 0.431(6) | 0.744(9) | 0.661(9) | 0.33(4) | 6.64 | 8 |
| miR-184 | 0.379(5) | 0.665(8) | 0.52(5) | 0.435(7) | 6.88 | 7 |
| miR-277 | 0.511(8) | 0.408(6) | 0.779(10) | 0.581(8) | 7.2 | 9 |
| miR-11 | 0.650(10) | 0.865(10) | 0.565(8) | 0.943(10) | 10 | 10 |
| miR-624 | 0.802(11) | 1.438(11) | 1.341(11) | 1.589(11) | 11 | 11 |
| Cyantraniliprole |  |  |  |  |  |  |
| miR-100-5p | 0.256(1) | 0.107(1) | 0.556(7) | 0.54(1) | 1.63 | 1 |
| miR-1-3p | 0.256(1) | 0.399(5) | 0.326(2) | 0.60(3) | 2.34 | 2 |
| miR-279d | 0.293(3) | 0.296(3) | 0.457(4) | 0.59(2) | 2.91 | 3 |
| miR-252a | 0.298(4) | 0.360(4) | 0.406(3) | 0.61(4) | 3.72 | 4 |
| miR-9a | 0.315(5) | 0.521(9) | 0.284(1) | 0.66(5) | 3.87 | 5 |
| U6 | 0.461(9) | 0.261(2) | 0.914(9) | 0.66(6) | 5.58 | 6 |
| miR-184 | 0.353(6) | 0.463(8) | 0.461(5) | 0.67(7) | 6.4 | 7 |
| miR-275 | 0.390(7) | 0.417(7) | 0.52(6) | 0.67(8) | 6.96 | 8 |
| miR-277 | 0.424(8) | 0.409(6) | 0.663(8) | 0.67(9) | 7.67 | 9 |
| miR-624 | 0.609(10) | 1.044(10) | 1.405(10) | 1.16(10) | 10 | 10 |
| miR-11 | 0.741(11) | 1.265(11) | 1.746(11) | 1.33(11) | 11 | 11 |
| Afidopyropen |  |  |  |  |  |  |
| miR-1-3p | 0.196(1) | 0.034(1) | 0.312(2) | 0.68(2) | 1.41 | 1 |
| miR-100-5p | 0.196(1) | 0.098(3) | 0.449(6) | 0.67(1) | 2.06 | 2 |
| U6 | 0.257(3) | 0.057(2) | 0.339(3) | 0.68(3) | 2.71 | 2 |
| miR-275 | 0.339(4) | 0.392(5) | 0.369(5) | 0.79(5) | 4.73 | 4 |
| miR-279d | 0.550(8) | 0.778(8) | 0.257(1) | 0.97(8) | 4.76 | 7 |
| miR-252a | 0.389(5) | 0.238(4) | 0.535(7) | 0.75(4) | 4.86 | 5 |
| miR-184 | 0.429(6) | 0.432(6) | 0.364(4) | 0.79(6) | 5.42 | 6 |
| miR-277 | 0.477(7) | 0.449(7) | 0.705(9) | 0.84(7) | 7.45 | 8 |
| miR-9a | 0.666(9) | 1.269(10) | 0.692(8) | 1.33(10) | 9.21 | 9 |
| miR-624 | 0.800(10) | 1.129(9) | 1.399(10) | 1.26(9) | 9.49 | 10 |
| miR-11 | 0.936(11) | 1.485(11) | 1.651(11) | 1.55(11) | 11 | 11 |
| Avermectin |  |  |  |  |  |  |
| miR-100-5p | 0.304(3) | 0.090(1) | 0.576(4) | 0.57(1) | 1.86 | 1 |
| miR-9a | 0.213(1) | 0.106(2) | 0.783(8) | 0.575(2) | 2.38 | 2 |
| U6 | 0.213(1) | 0.253(4) | 0.939(9) | 0.64(4) | 3.46 | 4 |
| miR-275 | 0.338(4) | 0.250(3) | 0.661(6) | 0.63(3) | 3.83 | 3 |
| miR-1-3p | 0.378(5) | 0.379(5) | 0.398(2) | 0.65(5) | 3.98 | 5 |
| miR-279d | 0.539(8) | 0.703(9) | 0.262(1) | 0.85(8) | 4.9 | 6 |
| miR-277 | 0.409(6) | 0.404(6) | 0.739(7) | 0.70(6) | 6.24 | 6 |
| miR-252a | 0.481(7) | 0.543(7) | 0.626(5) | 0.79(7) | 6.44 | 8 |
| miR-184 | 0.593(9) | 0.849(10) | 0.426(3) | 0.95(10) | 7.21 | 9 |
| miR-11 | 0.654(10) | 0.697(8) | 1.123(10) | 0.90(9) | 9.21 | 10 |
| miR-624 | 0.781(11) | 1.296(11) | 1.802(11) | 1.35(11) | 11 | 11 |
| Deltamethrin |  |  |  |  |  |  |
| miR-1-3p | 0.341(1) | 0.100(1) | 0.674(5) | 0.54(1) | 1.5 | 1 |
| miR-100-5p | 0.341(2) | 0.273(2) | 0.85(7) | 0.58(2) | 2.3 | 2 |
| miR-279d | 0.442(5) | 0.284(3) | 0.569(2) | 0.605(4) | 3.31 | 3 |
| U6 | 0.387(3) | 0.361(5) | 1.001(9) | 0.603(3) | 4.49 | 4 |
| miR-184 | 0.414(4) | 0.337(4) | 0.736(6) | 0.62(5) | 4.68 | 4 |
| miR-275 | 0.553(8) | 0.479(6) | 0.65(4) | 0.70(6) | 5.83 | 6 |
| miR-252a | 0.712(11) | 0.943(11) | 0.256(1) | 1.04(11) | 6.04 | 7 |
| miR-11 | 0.640(10) | 0.661(10) | 0.637(3) | 0.83(10) | 7.4 | 7 |
| miR-277 | 0.485(6) | 0.611(8) | 1.09(11) | 0.76(7) | 7.8 | 7 |
| miR-9a | 0.515(7) | 0.587(7) | 1.002(10) | 0.76(7) | 7.91 | 7 |
| miR-624 | 0.594(9) | 0.637(9) | 0.953(8) | 0.80(9) | 8.74 | 11 |
| β-cypermethrin |  |  |  |  |  |  |
| miR-1-3p | 0.265(3) | 0.065(1) | 0.382(2) | 0.61(1) | 1.57 | 1 |
| U6 | 0.207(1) | 0.157(2) | 0.56(7) | 0.62(2) | 2.3 | 2 |
| miR-9a | 0.207(1) | 0.202(4) | 0.527(6) | 0.64(3) | 2.91 | 3 |
| miR-100-5p | 0.287(4) | 0.194(3) | 0.408(3) | 0.641(4) | 3.46 | 4 |
| miR-279d | 0.408(6) | 0.383 (5) | 0.181(1) | 0.72 (5) | 3.5 | 5 |
| miR-184 | 0.446(7) | 0.475(7) | 0.515(4) | 0.77(7) | 6.09 | 6 |
| miR-277 | 0.348(5) | 0.457(6) | 0.661(9) | 0.75(6) | 6.34 | 7 |
| miR-11 | 0.621(9) | 0.878(9) | 0.52(5) | 1.07(9) | 7.77 | 8 |
| miR-275 | 0.517(8) | 0.608(8) | 0.779(10) | 0.86(8) | 8.46 | 9 |
| miR-252a | 0.723(10) | 1.135(10) | 0.565(8) | 1.23(10) | 9.46 | 10 |
| miR-624 | 0.847(11) | 1.325(11) | 1.341(11) | 1.40(11) | 11 | 11 |
| Insecticides |  |  |  |  |  |  |
| miR-100-5p | 0.312(1) | 0.219(2) | 0.478(2) | 0.72(1) | 1.41 | 1 |
| miR-1-3p | 0.351(3) | 0.202(1) | 0.387(1) | 0.73(2) | 1.57 | 2 |
| miR-277 | 0.312(1) | 0.312(3) | 0.551(4) | 0.76(3) | 2.45 | 3 |
| miR-184 | 0.383(4) | 0.451(5) | 0.485(3) | 0.79(5) | 4.16 | 4 |
| U6 | 0.416(5) | 0.358(4) | 0.579(5) | 0.77(4) | 4.47 | 5 |
| miR-279d | 0.467(6) | 0.626(6) | 0.655(6) | 0.89(6) | 6 | 6 |
| miR-9a | 0.549(7) | 0.812(8) | 0.674(7) | 1.03(8) | 7.48 | 7 |
| miR-275 | 0.62(8) | 0.733(7) | 0.73(9) | 1.01(7) | 7.71 | 8 |
| miR-252a | 0.719(9) | 0.852(9) | 0.69(8) | 1.12(9) | 8.74 | 9 |
| miR-624 | 0.849(10) | 1.179(10) | 0.976(10) | 1.36(10) | 10 | 10 |
| miR-11 | 0.976(11) | 1.413(11) | 1.151(11) | 1.55(11) | 11 | 11 |
| Developmental stages |  |  |  |  |  |  |
| miR-624 | 0.33(1) | 1.153(6) | 0.167(1) | 1.51(4) | 2.21 | 1 |
| miR-9a | 1.057(6) | 0.185(1) | 0.966(5) | 1.3(1) | 2.34 | 3 |
| miR-11 | 0.417(3) | 0.89(3)8 | 0.336(3) | 1.42(2) | 2.71 | 2 |
| miR-252a | 0.33(1) | 1.234(7) | 0.314(2) | 1.57(5) | 2.89 | 4 |
| miR-275 | 0.932(5) | 0.705(2) | 1.131(6) | 1.45(3) | 3.66 | 5 |
| U6 | 0.664(4) | 1.563(9) | 0.575(4) | 1.83(9) | 6 | 6 |
| miR-279d | 1.157(7) | 1.006(5) | 1.138(7) | 1.58(6) | 6.19 | 6 |
| miR-1-3p | 1.285(8) | 1.002(4) | 1.556(8) | 1.58(6) | 6.51 | 8 |
| miR-184 | 1.394(9) | 1.269(8) | 1.709(9) | 1.72(8) | 8.49 | 9 |
| miR-277 | 1.53(10) | 1.816(10) | 2.104(10) | 2.08(10) | 10 | 10 |
| miR-100-5p | 1.666(11) | 2.016(11) | 2.535(11) | 2.28(11) | 11 | 11 |
| Gender |  |  |  |  |  |  |
| miR-100-5p | 0.317(1) | 0.158(2) | 0.71(8) | 0.91(2) | 2.38 | 1 |
| miR-1-3p | 0.395(3) | 0.196(4) | 0.53(4) | 0.87(1) | 2.63 | 1 |
| miR-252a | 0.455(5) | 0.108(1) | 0.695(7) | 0.92(4) | 3.44 | 3 |
| miR-9a | 0.317(1) | 0.158(2) | 0.753(10) | 0.94(5) | 3.5 | 4 |
| miR-275 | 0.434(4) | 0.228(5) | 0.683(6) | 0.91(2) | 4.36 | 4 |
| miR-11 | 0.589(8) | 0.649(8) | 0.276(1) | 1.02(8) | 4.76 | 6 |
| miR-279d | 0.556(7) | 0.657(9) | 0.387(2) | 1.01(7) | 5.45 | 8 |
| miR-184 | 0.494(6) | 0.401(6) | 0.593(5) | 0.97(6) | 5.73 | 6 |
| miR-624 | 0.615(9) | 0.532(7) | 0.439(3) | 1.04(9) | 6.42 | 9 |
| miR-277 | 0.725(10) | 1.35(10) | 0.734(9) | 1.49(10) | 9.74 | 10 |
| U6 | 1.238(11) | 3.521(11) | 3.749(11) | 3.55(11) | 11 | 11 |
| Adult tissues |  |  |  |  |  |  |
| miR-624 | 0.295(1) | 0.98(4) | 0.197(1) | 1.55(4) | 2 | 1 |
| miR-275 | 0.546(4) | 0.37(2) | 0.472(4) | 1.39(1) | 2.38 | 2 |
| miR-252 | 0.638(5) | 0.277(1) | 0.724(5) | 1.42(2) | 2.66 | 3 |
| miR-11 | 0.295(1) | 1.157(5) | 0.241(2) | 1.63(5) | 2.66 | 3 |
| miR-100-5p | 0.457(3) | 0.8(3) | 0.318(3) | 1.49(3) | 3 | 5 |
| miR-9a | 0.964(6) | 1.302(6) | 1.306(7) | 1.9(6) | 6.24 | 6 |
| miR-1-3p | 1.511(9) | 1.417(7) | 1.986(8) | 1.91(7) | 7.71 | 7 |
| miR-184 | 1.273(7) | 1.486(8) | 2.109(9) | 1.92(8) | 7.97 | 8 |
| miR-277 | 1.422(8) | 1.581(9) | 2.163(10) | 1.98(9) | 8.97 | 9 |
| U6 | 1.686(10) | 2.417(11) | 1.26(6) | 2.58(10) | 9.01 | 10 |
| miR-279d | 1.85(11) | 2.385(10) | 2.477(11) | 2.59(11) | 10.74 | 11 |
